# Genome-wide association study for female fertility in Nordic Red cattle

**DOI:** 10.1186/s12863-015-0269-x

**Published:** 2015-09-15

**Authors:** Johanna K. Höglund, Bart Buitenhuis, Bernt Guldbrandtsen, Mogens S. Lund, Goutam Sahana

**Affiliations:** Center for Quantitative Genetics and Genomics, Department of Molecular Biology and Genetics Aarhus University, P.O. Box 50, DK 8830 Tjele, Denmark; Present address: Department of Animal Science, Aarhus University, P.O. Box 50, DK-8830 Tjele, Denmark

**Keywords:** Nordic Red Cattle, Female fertility index, Sequence analysis, Association study

## Abstract

**Background:**

The Nordic Red Cattle (**NRC**) consists of animls belonging to the Danish Red, Finnish Ayrshire, and Swedish Red breeds. Compared to the Holstein breed, NRC animals are smaller, have a shorter calving interval, lower mastitis incidence and lower rates of stillborn calves, however they produce less milk, fat and protein. Female fertility is an important trait for the dairy cattle farmer. Selection decisions in female fertilty in NRC are based on the female fertility index (**FTI**). FTI is a composite index including a number of sub-indices describing aspects of female fertility in dairy cattle. The sub-traits of FTI are: number of inseminations per conception (**AIS**) in cows (**C**) and heifers (**H**), the length in days of the interval from calving to first insemination (**ICF**) in cows, days from first to last insemination (**IFL**) in cows and heifers, and 56-day non-return rate (**NRR**) in cows and heifers. The aim of this study was first to identify QTL for FTI by conducting a genome scan for variants associated with fertility index using imputed whole genome sequence data based on 4207 Nordic Red sires, and subsequently analyzing which of the sub-traits were affected by each FTI QTL by associating them with the sub-traits.

**Results:**

A total 17,388 significant SNP markers (−log_10_(*P*) > 8.25) were detected for FTI distributed over 25 chromosomes. The chromosomes with the most significant markers were tested for associations with the underlying sub-traits: BTA1 (822 SNP), BTA2 (220 SNP), BTA3 (83 SNP), BTA5 (195 SNP), two regions on BTA6 (503 SNP), BTA13 (980 SNP), BTA15 (23 SNP), BTA20 (345 SNP), and BTA24 (104 SNP). The fertility traits underlying the FTI peak area were: BTA1 (IFLC, IFLH), BTA2 (AISH, IFLH, NRRH), BTA3 (AISH, NRRH), BTA5 (AISC, AISH, IFLH), BTA6 (region 1: AISH, NRRH; region 2: AISH, IFLH), BTA13 (IFLH, IFLC), BTA15 (IFLC, NRRH), and BTA24 (AISH, IFLH). For BTA20 all sub-traits had SNP markers with a –log_10_(*P*) > 10. Furthermore the genes assigned to the most significant SNP for FTI were located on BTA6 (*GPR125*), BTA13 (*ANKRD60*), BTA15 (*GRAMD1B*), and BTA24 (*ZNF521*).

**Conclusion:**

This study 1) shows that many markers within FTI QTL regions were significantly associated with both AISH and IFLH, and 2) identified candidate genes for FTI located on BTA6 (*GPR125*), BTA13 (*ANKRD60*), BTA15 (*GRAMD1B*), and BTA24 (*ZNF521*). It is not known how the genes/variants identified in this study regulate female fertility, however the majority of these genes were involved in protein binding, 3) a SNP in a QTL region for FTI on BTA20 was previously validated in three cattle breeds.

**Electronic supplementary material:**

The online version of this article (doi:10.1186/s12863-015-0269-x) contains supplementary material, which is available to authorized users.

## Background

The Nordic Red Cattle (**NRC**) consists of animals belonging to the Danish Red, Finnish Ayrshire, and Swedish Red breeds. Compared to the Holstein which is the dominating dairy cattle breed in the Denmark, NRC animals are smaller, have a shorter calving interval, lower mastitis incidence and lower rates of stillborn calves. However, they also produce less milk, fat and protein [1]. Female fertility is an important trait for the dairy cattle farmer. Poor fertility leads to extra inseminations, higher veterinary costs, and higher culling rate causing higher replacement costs. Impaired female fertility is the major reason for culling in the dairy industry [2]. In the Nordic countries genetic improvement of female fertility in the NRC cattle population is achieved by including a fertility index (**FTI**) in the breeding goal. FTI reflects how easily a cow or a heifer conceives given the index is composed of four different female fertility traits namely: number of inseminations per conception, interval from calving to first insemination, days from first to last insemination, and 56-day non-return rate.

Genetic evaluation for three NRC populations from Denmark, Sweden and Finland are carried out jointly by Nordic Cattle Genetic Evaluation (http://www.nordicebv.info). The trait definitions have been standardized across the three countries and the cattle industry has made the large NRC dataset available for cattle genetic research.

Previous genetic analyses of female fertility in NRC showed that the heritability is in the range of 0.02 to 0.04 [1]. A trait with low heritability requires large progeny groups in order to attain an acceptable level of accuracy for the breeding value. International cooperation is therefore beneficial as a large number of registrations increase the accuracy of the estimated breeding values.

Previous studies on the identification of DNA markers have been focusing on the individual red breeds [3, 4], however a joint association analysis including the three NRC populations is lacking. Furthermore, a limitation of these previous studies [3, 4] is that the marker panels used only represent a small fraction of the variants segregating in the population. With the availability of data from the three countries and the whole genome sequence (**WGS**), a joint analysis could improve the power to identify the causal mutation [5]. The power to detect QTL increases because there is an improvement of accuracy of the breeding value by pooling the data from the three countries together. Furthermore using WGS will capture the linkage disequilibrium structure of the population better due to the higher marker density and in addition WGS includes some of the causal mutations underlying FTI. The identified DNA markers influencing female fertility traits in NRC [4–6] can potentially be used for routine genomic evaluation where additional weight can be put on certain genomic regions/variants [7].

The joint association analysis of the NRC population with imputed WGS data allows the detection of QTL for FTI and the determination of which sub-traits are affected by these QTL for FTI. Therefore our first objective of this study was to identify QTL for FTI by conducting a genome scan for variants associated with FTI in the joint NRC population using imputed WGS. The second objective was to re-analyze the identified QTL region for FTI to ascertain which sub-trait(s) of FTI are influenced by these QTL.

## Methods

The analysis was based on a two-step approach. In the first step the genome was screened for the fertility index only based on a sire model without considering relationship among sires. In the second step targeted regions were selected based on the genome scan and re-analyzed for the individual female fertility traits using an animal model considering relationship among all animals in the study.

### Animals and phenotype data

No animal experiments were performed in this study, and, therefore, approval from the ethics committee was not required. A total of 4207 Nordic Red sires from Denmark (RDCDNK), Sweden (RDCSWE) and Finland (RDCFIN) with official breeding values for female fertility traits were used for the association study. Estimated breeding values (**EBV**s) were predicted by the Nordic Cattle Genetic Evaluation (**NAV**) for each animal as part of routine genetic evaluation of Nordic dairy cattle breeds. Predictions were based on first to third parity records. BLUP EBVs were predicted using multi-trait sire models (**SM**). For details regarding the phenotypes recorded and models used in routine breeding value prediction, see http://www.nordicebv.info. The reliability of the breeding value is in the range of 40 to 99 % (Additional file 1: Figure S1).

Female fertility index reflects the ease with which a heifer or cow was able to conceive. FTI is a compound index and its sub-traits are: number of inseminations per conception (**AIS**) in cows and heifers, the length in days of the interval from calving to first insemination (**ICF**) in cows, and days from first to last insemination (**IFL**) in cows and heifers. In addition 56-day non-return rate (**NRR**) in cows and heifers were analyzed even though it had no weight in the FTI. The reason to include NRR in our analyses was that NRR is a trait of biological importance understanding female fertility. For AIS, IFL and NRR EBVs were predicted separately for 1st parity animals (heifers, suffixed **H**) and 2nd and 3rd parity animals (cows, suffixed **C**). For more information about the heritability and correlations between the traits please see http://www.nordicebv.info/NR/rdonlyres/5CD2E4DC-F82A-4809-A770-3022E270E205/0/PrinciplesNyeste.pdf.

In step I FTI was analyzed for its association with SNPs. In step II selected QTL regions identified for FTI were targeted and analyzed for individual female fertility traits: AIS, IFL, ICF and NRR as well as for FTI with an animal model considering the relationship among all the animals in the study.

### Whole genome sequence data

The 253 dairy cattle sequences originating from a combination of sequences processed at Aarhus University [8] and sequences from the 1000 Bull Genomes Project dataset *Run2* [9] were available. The sequencing of Nordic bulls (Jersey, Holstein, Nordic Red Cattle) at Aarhus University, Foulum was done using Illumina sequencers at Beijing Genomics Institute, Shenzhen, China and at Aarhus University. Sequencing was shotgun paired-end sequencing. Where necessary, fastq data were converted from Illumina to Sanger quality encoding using a patched version of *maq* [10]. They were aligned to the UMD3.1 assembly of the cattle genome [11] using *bwa* [12]. They were converted to raw BAM files using *samtools* [10]. Quality scores were re-calibrated using the Genome Analysis Toolkit [13] following the Human 1000 Genome guidelines incorporating information from dbSNP [14]. Sequences were realigned around insertion/deletions using the Genome Analysis Toolkit. Variants were called using the Genome Analysis Toolkit’s *UnifiedGenotyper*. Genomes for the 1000 Bull Genomes Project were sequenced in a number of laboratories and collected at the Department of Primary Industries, Victoria, Australia [9]. The breeds included in the sequencing project can be found at: http://www.1000bullgenomes.com/. Sequences were aligned to the same reference genome as used at Aarhus University. Variant calling was done using samtools’ *mpileup* function. Variant Call Files from Aarhus University and the 1000 Bull Genomes project were combined using the Genome Analysis Toolkit’s *CombineVariants* with precedence given to calls from the Nordic dataset for animals appearing in both datasets.

### Imputation HD and sequence data

Imputation of 50 K SNP to the full sequence was done in two steps. First, in another study (N.K. Kadri, *pers. comm*.) the 50 K genotypes for 12,322 Nordic bulls (from Nordic Holstein, RDC and Danish Jersey) were imputed to HD genotypes using the software IMPUTE2 [14]. A reference containing HD genotypes for 2036 bulls (902 Holstein, 735 Nordic Red and 399 Danish Jersey) was available.

Second, the 12,322 bulls imputed to HD genotypes were further imputed to the full sequence variants. Variants from the 253 dairy bulls were used as reference to impute the imputed HD data to the whole genome level using the software Beagle [15]. For imputation from HD to sequence data, chromosomes were divided into chunks of about 20,000 consecutive markers with an overlap of 250 markers at each end to minimize imputation error at ends of the chunks. Only polymorphisms identified both at Aarhus University dataset and the 1000 Bull Genomes dataset were imputed. For positions containing both a SNP and an insertion-deletion polymorphism (INDEL), the INDEL was deleted. SNPs at positions with disagreements between alleles from sequence and HD data were deleted. The reference data was pre-phased with Beagle v3.3.2 [15] and all markers with an r^2^ value below 0.9 were removed. This left a total of 8,938,927 markers for chromosomes 1–29.

### Statistical method for association analysis

#### Step I: Genome scan for FTI

A sire model was used for the initial genome screening for markers associated with FTI. A SNP-by-SNP analysis was carried out in which each SNP in turn was tested for association with the phenotype. The following model was used to estimate SNP effects:

$${y}_{ijk}=\mu +{p}_i+b{x}_{ijk}+{s}_j+{e}_{ijk}$$

Where *y*_*ijk*_ was the EBV of individual *k* belonging to the half-sib (sire) family *j* from population *i*, *μ* was the general mean, *p*_*i*_ is fixed effect of *i*‘th population (RDCDNK, RDCSWE and RDCFIN), *b* was the allelic substitution effect, *x*_*ijk*_ was the number of copies of an allele (with an arbitrary labeling) of the SNP count in the individual *k* (corresponding to 0, 1, or 2 copies), and *s*_*j*_ was the random effect of the *j*‘th half-sib family assumed to follow a normal distribution *s* ~ *N*(0, *σ*_*s*_^2^) where *σ*_*s*_^2^ was the sire variance, and *e*_*ijk*_ was a random residual of individual *k* assumed to follow a normal distribution *e* ~ *N*(0, *σ*_*e*_^2^). All statistical analyses were conducted using DMU [16]. The null hypothesis H_0_: b = 0 was tested with a *t*-test. A SNP was considered significant if the –log_10_(*P*) value for the SNP exceeded a genome wide Bonferroni corrected threshold of 8.25 (= −log_10_(0.05/8,938,927)) corresponding to a nominal type I error rate of 5 % corrected for 8,938,927 simultaneous tests.

#### Step II: Association analyses for the targeted regions

**Fig. 1:**
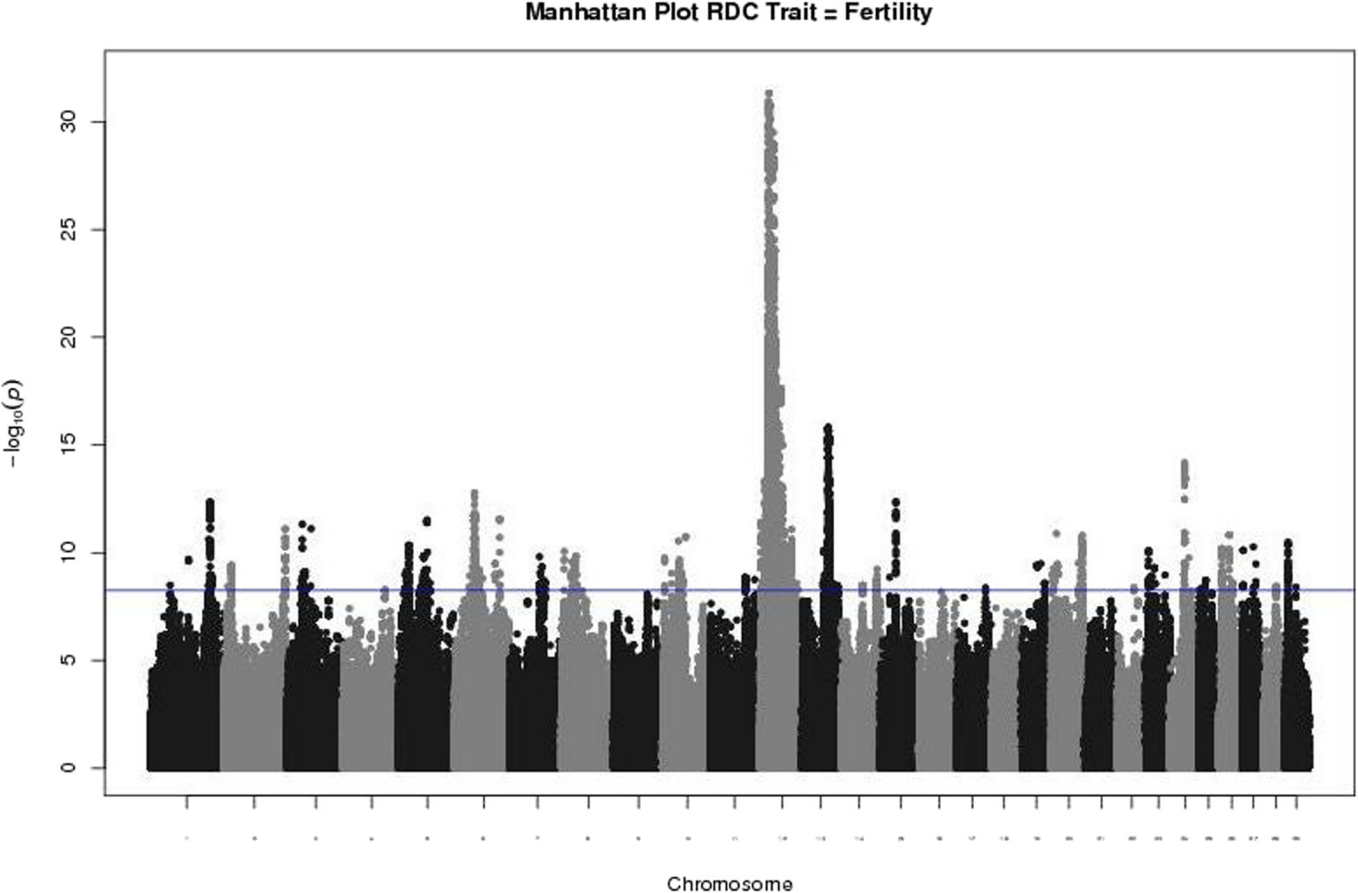
Manhattan plot of the association results of Fertility index in Nordic Red Cattle. On the x-axis the chromosomes are represented. On the y-axis the –log_10_(*P*) is presented. The horizontal blue line indicates Bonferroni corrected *P*-value at the 5 % level

The results from the step I analysis were plotted in a Manhattan plot (Fig. 1). QTL peaks included in step II analyses were identified subjectively based on the Manhattan plot. The most significant SNP was the marker with the largest –log_10_(*P*) value in that peak. QTL regions were defined as a section of chromosome 0.5 Mb on both sides of the most significant SNPs for FTI in the genome. The selected regions were screened for association with the sub-indices. An animal model considering all relationships among individuals was used.

The association between the SNP and the phenotype was assessed by a single-locus regression analysis for each SNP separately, using a linear mixed model [17]. The model was as follows:

$${y}_{ij}=\mu +{p}_i+b{x}_{ij}+{u}_{ij}+{e}_{ij}$$

where *y*_*ij*_ was the EBV for *j*‘th bull from *i*‘th population, *μ* was the overall mean, *p*_*i*_ was a fixed effect of *i*‘th population (RDCDNK, RDCSWE and RDCFIN), *b* was the allelic substitution effect, *x*_*ij*_ was the number of copies of an allele (with an arbitrary labeling) of the SNP count in the individual *j* (corresponding to 0, 1, or 2 copies), *u*_*ij*_ was the random polygenic effects. The vector ***u*,** whose elements were the *u*_*ij*_ was assumed to follow a multivariate normal distribution ***u*** ~ N (**0**, ***A*** *σ*_*u*_^*2*^) where ***A*** was the additive relationship matrix between sires and *σ*_*u*_^*2*^ was the polygenic variance, and *e*_*j*_ was the random residual of individual *j* with a normal distribution N(0,*σ*_*e*_^*2*^). The model was fitted by REML using the software DMU [16]. The estimates and standard errors of the fixed effects estimates were obtained from DMU. Testing for the presence of an effect of a marker was done using a *t*-test against a null hypothesis of H_0_: *b* = 0. A SNP was considered significant if the –*log*(*p*) value for the SNP exceeded a genome wide Bonferroni corrected threshold of 8.25 (= −log_10_(0.05/8,938,927)) corresponding to a nominal type I error rate of 5 % corrected for 8,938,927 simultaneous tests.

#### Annotation of the top SNP

All positions provided in this text refer to the UMD3.1 assembly. The annotations of the most significant SNP markers in the selected regions were extracted from the ENSEMBL data base (http://www.ensembl.org, [18]): ENSEMBL *Bos taurus* 75.31, UMD3.1.

## Results

### Step I

The results of the step I analysis for FTI are presented Fig. 1 and in Additional file 2: Table S1. In total 17,388 significant SNP markers (−log_10_(*P*) > 8.25) were detected for FTI distributed over 25 different chromosomes. The most significant QTL was identified on BTA12 with 12,860 significantly associated SNP. For this QTL the causal variant, a 660-Kb deletion around 20.5 Mb at BTA12, has previously been described [5]. The other QTL were located on BTA1 (822 SNP), BTA2 (219 SNP), BTA3 (83 SNP), BTA5 (195 SNP), BTA6 (503 SNP), BTA7 (146 SNP), BTA8 (117 SNP), BTA10 (294 SNP), BTA11 (215 SNP), BTA13 (980 SNP), BTA14 (43 SNP), BTA15 (23 SNP), BTA17 (1 SNP), BTA19 (47 SNP), BTA20 (345 SNP), BTA22 (2 SNP), BTA23 (38 SNP), BTA24 (104 SNP), BTA25 (53 SNP), BTA26 (224 SNP), BTA27 (15 SNP), BTA28 (9 SNP) and BTA29 (46 SNP). The most significant QTL peaks are presented in Table 1.

**Table 1:** Overview of the genome-wide significant QTL for fertility index in Nordic Red cattle analyzed with a sire model

Chromosome	Start position (bp)	End position (bp)	Position of the most significantly associated SNP (bp)	Marker name (rsname)	MAF	−log_10_(*P*)	Allele substitution effect	Standard errors
1	128,128,973	129,128,826	128,628,880	rs133429171	0.31	12.35	1.70	0.23
2	132,612,052	133,611,970	133,111,977	rs109408437	0.44	11.10	−1.46	0.21
3	32,891,184	33,888,417	33,388,881	rs43336559	0.48	11.31	−1.49	0.22
5	61,407,644	62,407,443	61,907,474	rs110369759	0.28	11.50	1.70	0.24
6	43,012,003	43,998,190	43,511,992	rs41983284	0.40	12.75	1.63	0.22
6	97,617,381	98,613,547	98,115,824	rs208894094	0.36	11.53	−1.59	0.23
13	56,548,495	57,548,174	57,048,185	rs133575483	0.26	15.82	−2.02	0.24
15	34,177,504	35,177,124	34,677,367	rs41763261	0.21	12.37	−1.91	0.26
20	66,626,286	67,624,451	67,124,570	rs42467155	0.39	10.81	1.55	0.23
24	31,321,194	32,320,535	31,820,659	rs137134841	0.43	14.18	−1.73	0.22

### Step II

The regions selected based on association results for FTI (Table 1) were reanalyzed for the individual female fertility traits using sequence data in an animal model (Table 2). The QTL shown in Table 2 showed significant association with fertility sub-traits, however the magnitude of test statistics varied for individual sub-traits.

**Table 2:** Most significant SNP marker for FTI and the underlying female fertility traits

Trait name^a^	Marker name	rsname	−log_10_(*P*)	Annotation^b^	Ensemble	Gene name	Gene description	Proportion of additive genetic variance explained by the SNP
BTA1								
FTI	Chr1:128614529	rs137748444	11.49	intergenic_variant				1.17
AISC	Chr1:127295226	rs43271631	9.89	intron_variant	ENSBTAG00000002232	TRPC1	*Bos taurus* transient receptor potential cation channel, subfamily C, member 1	0.89
AISH	Chr1:128189301	rs136231770	11.75	upstream_gene_variant	ENSBTAG00000021886	GRK7	*Bos taurus* G protein-coupled receptor kinase 7	1.21
ICF	Chr1:128663781	Chr1:128663781^c^	7.32	intergenic_variant				0.67
IFLC	Chr1:127295226	rs43271631	12.24	intron_variant	ENSBTAG00000002232	TRPC1	*Bos taurus* transient receptor potential cation channel, subfamily C, member 1	1.26
IFLH	Chr1:128189301	rs136231770	12.49	upstream_gene_variant	ENSBTAG00000021886	GRK7	*Bos taurus* G protein-coupled receptor kinase 7	1.32
NRRC	Chr1:127295226	rs43271631	7.37	intron_variant	ENSBTAG00000002232	TRPC1	*Bos taurus* transient receptor potential cation channel, subfamily C, member 1	0.72
NRRH	Chr1:128106801	rs43262199	8.36	intergenic_variant				0.93
BTA2								
FTI	Chr2:133075293	rs42761190	8.51	intergenic_variant				0.90
AISC	Chr2:133079214	rs42761183	6.17	intergenic_variant				0.57
AISH	Chr2:132321329	rs209242168	13.30	intron_variant	ENSBTAG00000040215	EIF4G3	Uncharacterized protein	1.44
ICF	Chr2:133126588	rs109245794	9.98	upstream_gene_variant	ENSBTAG00000012217	PLA2G2F	*Bos taurus* phospholipase A2, group IIF	0.95
IFLC	Chr2:133077462	rs42761186	6.00	intergenic_variant				0.57
IFLH	Chr2:132321329	rs209242168	10.50	intron_variant	ENSBTAG00000040215	EIF4G3	Uncharacterized protein	1.13
NRRC	Chr2:131498393	rs110082997	6.92	intergenic_variant				0.74
NRRH	Chr2:132321329	rs209242168	13.01	intron_variant	ENSBTAG00000040215	EIF4G3	Uncharacterized protein	1.51
BTA3								
FTI	Chr3:33388881	rs43336559	14.22	intergenic_variant				1.56
AISC	Chr3:33388881	rs43336559	15.78	intergenic_variant				1.47
AISH	Chr3:30770425	rs134664378	23.07	intron_variant	ENSBTAG00000014299	RHOC	*Bos taurus* ras homolog gene family, member C	2.48
ICF	Chr3:31574900	rs43334971	8.05	intron_variant				0.80
IFLC	Chr3:33388881	rs43336559	11.99	intergenic_variant				1.24
IFLH	Chr3:35629755	rs109158850	15.86	intron_variant	ENSBTAG00000031575	VAV3	vav 3 guanine nucleotide exchange factor	1.69
NRRC	Chr3:33388483	rs381207703	18.34	intergenic_variant				1.87
NRRH	Chr3:32024752	rs43332934	21.55	upstream_gene_variant	ENSBTAG00000014102	WDR77	*Bos taurus* WD repeat domain 77	2.41
BTA5								
FTI	Chr5:62781359	rs135099682	10.79	intergenic_variant				1.15
AISC	Chr5:62781359	rs135099682	9.99	intergenic_variant				0.89
AISH	Chr5:62781359	rs135099682	13.87	intergenic_variant				1.37
ICF	Chr5:61879166	rs207754946	8.01	intergenic_variant				0.76
IFLC	Chr5:61625072	rs434505378	10.16	intergenic_variant				0.97
IFLH	Chr5:62781359	rs135099682	13.45	intergenic_variant				1.34
NRRC	Chr5:64863598	rs383102267	6.74	intergenic_variant				0.59
NRRH	Chr5:61836879	rs211681535	6.95	intergenic_variant				0.69
BTA6								
FTI	Chr6:43511992	rs41983284	13.59	intron_variant	ENSBTAG00000004653	GPR125	G protein-coupled receptor 125	1.54
AISC	Chr6:43223916	rs133258175	12.17	intergenic_variant				1.15
AISH	Chr6:44642153	rs209918674	20.98	intergenic_variant				1.99
ICF	Chr6:42542483	rs110363606	7.77	intron_variant	ENSBTAG00000047743	KCNIP4	Bos taurus Kv channel interacting protein 4	0.71
IFLC	Chr6:45794025	rs109808198	12.17	intergenic_variant				1.26
IFLH	Chr6:41455222	rs43610453	16.21	intron_variant	ENSBTAG00000005108	SLIT2	Slit homolog 2 (Drosophila)	1.59
NRRC	Chr6:42245672	rs109518200	12.48	intron_variant	ENSBTAG00000047743	KCNIP4	*Bos taurus* Kv channel interacting protein 4	1.27
NRRH	Chr6:44642153	rs209918674	17.63	intergenic_variant				1.80
BTA6								
FTI	Chr6:98115824	rs208894094	11.29	intergenic_variant				1.26
AISC	Chr6:98115824	rs208894094	6.38	intergenic_variant				0.58
AISH	Chr6:99400480	rs379908987	14.72	intron_variant	ENSBTAG00000022449	SCD5	*Bos taurus* stearoyl-CoA desaturase 5	1.51
ICF	Chr6:98048673	rs133357086	10.91	intergenic_variant				1.06
IFLC	Chr6:98115824	rs208894094	8.88	intergenic_variant				0.92
IFLH	Chr6:99400480	rs379908987	13.58	intron_variant	ENSBTAG00000022449	SCD5	*Bos taurus* stearoyl-CoA desaturase 5	1.42
NRRC	Chr6:98385129	rs210763629	4.43	intergenic_variant				0.45
NRRH	Chr6:96953202	rs110379023	12.73	intron_variant	ENSBTAG00000035776	C4orf22	Chromosome 4 open reading frame 22	1.19
BTA13								
FTI	Chr13:58664049	rs378998625	14.85	intron_variant	ENSBTAG00000013574	ANKRD60	Ankyrin repeat domain 60	1.75
AISC	Chr13:56125951	rs109185706	12.69	intergenic_variant				1.18
AISH	Chr13:59923876	rs41700956	16.72	intergenic_variant				2.28
ICF	Chr13:59112800	rs42555672	12.19	downstream_gene_variant	ENSBTAG00000013574	ANKRD60	Ankyrin repeat domain 60	1.26
IFLC	Chr13:57020976	rs109484180	13.17	intergenic_variant				1.37
IFLH	Chr13:59923876	rs41700956	16.77	intergenic_variant				2.29
NRRC	Chr13:60559073	rs41699546	6.44	intergenic_variant				0.60
NRRH	Chr13:57048934	rs110280398	12.14	intergenic_variant				1.23
BTA15								
FTI	Chr15:34677367	rs41763261	15.90	intron_variant	ENSBTAG00000001410	GRAMD1B	GRAM domain containing 1B	1.68
AISC	Chr15:34737376	rs110670590	10.87	intron_variant	ENSBTAG00000001410	GRAMD1B	GRAM domain containing 1B	0.99
AISH	Chr15:33428419	rs382278362	9.99	intergenic_variant				0.97
ICF	Chr15:34702074	rs41763326	9.86	intron_variant	ENSBTAG00000001410	GRAMD1B	GRAM domain containing 1B	0.89
IFLC	Chr15:34677367	rs41763261	15.55	intron_variant	ENSBTAG00000001410	GRAMD1B	GRAM domain containing 1B	1.55
IFLH	Chr15:33428419	rs382278362	10.81	intergenic_variant				1.08
NRRC	Chr15:34829707	rs378927222	11.19	intergenic_variant				1.67
NRRH	Chr15:34802991	rs208575577	11.65	intergenic_variant				1.50
BTA20								
FTI	Chr20:67116858	rs133488500	11.44	intergenic_variant				1.39
AISC	Chr20:67623217	rs110045690	13.52	intergenic_variant				1.22
AISH	Chr20:68086468	rs208936479	15.87	intergenic_variant				1.68
ICF	Chr20:66806800	rs384363430	10.20	intergenic_variant				0.93
IFLC	Chr20:67124570	rs42467155	13.09	intergenic_variant				1.50
IFLH	Chr20:68084517	rs383590013	15.71	intergenic_variant				1.71
NRRC	Chr20:67623217	rs110045690	10.79	intergenic_variant				1.05
NRRH	Chr20:68086468	rs208936479	12.82	intergenic_variant				1.46
BTA24								
FTI	Chr24:31820659	rs137134841	18.50	intron_variant	ENSBTAG00000007383	ZNF521	Zinc finger protein 521	2.17
AISC	Chr24:31820659	rs137134841	15.00	intron_variant	ENSBTAG00000007383	ZNF521	Zinc finger protein 521	1.48
AISH	Chr24:31817915	rs381174897	25.32	intron_variant	ENSBTAG00000007383	ZNF521	Zinc finger protein 521	2.80
ICF	Chr24:31442858	rs43737972	7.94	intergenic_variant				0.70
IFLC	Chr24:31820659	rs137134841	14.49	intron_variant	ENSBTAG00000007383	ZNF521	Zinc finger protein 521	1.58
IFLH	Chr24:31817915	rs381174897	22.20	intron_variant	ENSBTAG00000007383	ZNF521	Zinc finger protein 521	2.50
NRRC	Chr24:30565598	rs42446963	11.49	intergenic_variant				1.16
NRRH	Chr24:31817915	rs381174897	18.03	intron_variant	ENSBTAG00000007383	ZNF521	Zinc finger protein 521	2.13

A marker is significant if the –log_10_(*P*) > 8.25

^a^FTI: fertility index, AIS: number of inseminations per conception, ICF: length in days of the interval from calving to first insemination, IFL: days from first to last insemination, and NRR: 56-day non-return rate (Suffix h: heifers and suffix c: cows)

^b^In case the SNP marker is annotated as a downstream_gene_variant or an upstream_gene_variant the gene closest located to this SNP is mentioned

^c^SNP marker did not have an rs id

On BTA1 the associations with the highest test statistic were found for IFLC and IFLH. The most significant SNP marker for IFLC was located in the *TRPC1* gene, while the most significant SNP marker for IFLH was annotated as an upstream gene variant of the *GRK7* gene. On BTA2 the associations with the highest test statistic were found for AISH, IFLH, and NRRH. The SNP BTA2:132321329, (rs209242168) was the SNP with the highest test statistics for all three traits. This SNP is located within the *EIF4G3* gene. On BTA3 the associations with the highest test statistic were found for AISH and NRRH. For the QTL for AISH the most significant SNP was located within the *RHOC* gene, while for the QTL for NRRH the most significant SNP was l annotated as an upstream gene variant of the *WDR77* gene. On BTA5 the SNP with the highest test statistic for FTI was BTA5:62781359 (rs135099682) with a –log_10_(*P*) of 10.79. The same marker was also the most significant marker for the traits AISC, AISH and IFLH with –log_10_(*P*) values of 9.99, 13.87, and 13.45 respectively.

On BTA6 the associations with the highest test statistic were found for AISH and NRRH. Both SNPs (Chr6:43223916, Chr6:44642153) were located in an intergenic region (Table 2). However the most significant SNP marker (BTA6:43511992, rs41983284) for FTI was located within the *GPR125* gene. A second QTL on BTA6 was detected and showed the strongest association with AISH and IFLH. The SNP BTA6:99400480 (rs379908987) was the most significant SNP for both AISH and IFLH and were located within the *SCD5* gene. The SNP with the highest test statistics for NRRH (BTA6:96953202, rs110379023) was located within the *C4orf22* gene. Furthermore, FTI was most strongly associated with the SNP BTA6:98115824 (rs208894094). This SNP was also the most significant marker for AISC and IFLC.

On BTA13 the associations with the highest test statistic were found for IFLC and IFLH, whereas the marker with the highest test statistic for FTI was located in the *ANKRD60* gene. On BTA15 the associations with the highest test statistic were found for IFLC and NRRH. The most significant SNP marker for IFLC is located within the *GRAMD1B* gene, while the most significant SNP marker for NRRH is located in an intergenic region. On BTA20 all individual female fertility traits had a –log_10_(*P*) above 10. All SNP markers detected were located in an intergenic region. On BTA24 the associations with the highest test statistic were found for AISH and IFLH. The significant SNPs were located within the *ZNF521* gene.

## Discussion

In this study we have applied a two-step approach to screen the cattle genome for QTL for the female fertility index. In the first step the cattle genome was screened for QTL for the FTI based on the whole genome sequence variants using a sire model without considering the relationship among all animals in the study except the sire-son relationship. This was done to pre-select markers to analyze with a linear mixed model. Based on the results in step I, targeted QTL regions for the FTI were reanalyzed for the underlying female fertility traits using sequence data and an animal model taking the relationship among sires included in the study. Below we discuss the findings from individual targeted regions.

### BTA1

In this region for the FTI QTL AISC, IFLC and NRRC have their most significant SNP (BTA1:127295226, rs43271631) in common. AISC and IFLC are phenotypes which are highly connected as they measure the same i.e., AIS reflects the number of inseminations, whereas IFL measures the time this has taken. BTA1:127295226 is located in intronic region of *TRPC1. TRPC1* has functions related to protein binding (GO:0005515). In mice it has been shown that osteoblast formation and function is regulated by a *TRPC1* protein-dependent pathway [19]. Furthermore, AISH and IFLH share their most significant SNP. It is located up-stream of *GRK7*. Höglund et al. [6] validated two SNP in Nordic Holstein, Jersey and Nordic Red for NRRH at 124.8 Mb and 135.6 Mb, which is 4 to 5 Mb away from the QTL peak for FTI detected in this study. In Danish and Swedish Holstein significant SNPs were detected at 72.0 and 92.4 Mb respectively [20].

### BTA2

The most significant marker (BTA2:132321329, rs209242168) was in common for the traits AISH, IFLH and NRRH and located in the intron of *EIF4G3*. The function of *EIF4G3* is related to protein binding (GO:0005515). Previously a SNP on BTA2 has been detected for NRRC at 12.4 Mb in Finnish Ayrshire [4]. Even though the Finnish Ayrshire is part of the Nordic Red cattle population the SNP at 12.4 Mb for NRRC did not show significant effect in our study.

### BTA3

The most significant SNP for FTI (BTA3:33388881, rs43336559) was also the most significant SNP for AISC and IFLC. This SNP is intergenic close to SLC6A17 gene (BTA3: 33,337,960-33,373,481). *SLC6A17* gene functions as a vesicular transporter selective for proline, glycine, leucine, and alanine [21]. Complex Vertebral Malformation (**CVM**), which is characterized by misshapen and fused vertebrae around the cervico-thoracic junction is caused by a mutation in the Golgi-resident nucleotide-sugar transporter encoded by *SLC35A3* located a 43.4 Mb on BTA3 [22]. This QTL on BTA3 has most significant effect on number of insemination, non-return rate and interval from first to last insemination indicating that it could be related to early embryonic death. Therefore *SLC6A17* is a strong candidate gene for this QTL. Sahana et al. [20] detected significant SNP markers for FTI at 81.3 Mb in Danish and Swedish Holstein. Höglund et al. [6] has validated 10 SNPs on BTA3 for FTI, AISH, NRRC, and NRRH. The closest marker is for FTI at 36.9 Mb, the other 9 markers were not in the range of 32.8 to 33.8 Mb.

### BTA5

The most significant SNP for FTI (BTA5:62781359, rs135099682) was also the most significant SNP for AISC, AISH, and IFLC. This SNP was not assigned to any gene. Sahana et al. [20] detected a significant SNP at 116.3 Mb in Nordic Holstein for FTI, AISC, and IFLC. Höglund et al. [6] have validated SNP for ICF at 88.1 Mb and at 15.2, 15.5, and 37.2 Mb for NRRH. None of these SNPs were located in the QTL region detected in this study.

### BTA6

BTA6 harbors 2 QTL for fertility traits 54 Mb apart. In the first QTL defined in the region 43.0 to 43.9 Mb the most significant marker (BTA6:43511992, rs41983284) was located in an intron in *GPR125. GPR125* has functions related to protein binding (GO:0005515). Even though many female fertility traits were highly significant, none of the traits shared their most significant SNP (Table 2). The most significant SNPs for ICF and NRRC were located in intron regions of *KCNIP4*. The function of *KCNIP4* is among others related to protein binding (GO:0005515). In the region from 97.6 to 98.6 Mb the most significant SNP marker (BTA6:98115824, rs208894094) for FTI was also the most significant marker for AISC and IFLC. Furthermore AISH and IFLH had their most significant marker (BTA6:99400480, rs379908987) located in *SDS5* gene. The function of *SDS5* is related to stearoyl-CoA desaturase activity (GO:0004768). Sahana et al. [20] identified a SNP marker for ICF on BTA6 in Danish and Swedish Holstein around 89.7 Mb. Pimentel et al. [23] identified a candidate gene (*IGFBP7*) located at BTA6 influencing NRRH and IFLH. However this gene is not located within any of the QTL areas.

### BTA13

The most significant SNP maker for FTI on BTA13 (BTA13:58664049, rs378998625) was located in the gene *ANKRD60*. This gene is related to protein binding (GO:0005515). In a window of 1 Mb around BTA13:58664049 the traits AISH, AISC, ICF, IFLH, IFLC and NRRH showed highly significant markers (Table 2). None of the top associated marker for the individual traits was in common except for AISH and IFLH (BTA13:59923876, rs41700956). The traits AISH and IFLH share the underlying biology as AISH reflect the number of inseminations and IFLH measures the time between first and last insemination. Previously, Schulman et al. [4] detected a significant SNP on BTA13 for ICF in Finnish Ayrshire which was located at 18.1 Mb. In Nordic Holstein (NH) two SNP markers were detected close to the region of 56.5 to 57.5 Mb [20] on BTA13 for ICF, but the major QTL for ICF in NH was in the region 33.9 to 34.0 Mb. Furthermore, a QTL affecting ICF on BTA13 in Nordic Holstein was validated in Jersey and NRC by Höglund et al. [6]. The 4 validated SNP markers were in the range of 28 to 45 Mb. However, none of the validated SNPs across three breeds for ICF were significantly associated with FTI in this study. As the peak detected for FTI as well as AISH, AISC, ICF, IFLH, IFLC and NRRH in NRC does not show overlap with QTL detected for fertility traits in other cattle breeds indicates that this QTL may be private to the NRC population.

### BTA15

In the region 34.1 to 35.1 Mb FTI, AISC, ICF, and IFLC did not have their most significant SNP in common, but each of their significant SNP was located in the intron region of *GRAMD1B*. This gene’s involvement with female fertility is unknown. Höglund et al. [6] confirmed one SNP marker influencing IFLH in three cattle breeds (Nordic Holstein, Jersey and Nordic Red), however this marker was located at 23.6 Mb and did not overlap with the QTL detected for FTI in this study.

### BTA20

The most significant SNP for FTI is BTA20:67116858 (rs133488500). All the female fertility traits have high –log_10_(*P*) of over 10 in the region of 500 kbp around SNP marker BTA20:67116858 (Table 2). None of these SNP markers were annotated to a gene. One marker (BTB-01617409) located at 66,926,929 bp on BTA20 has been validated in Nordic Holstein, Jersey and Nordic Red for FTI [6]. This marker is only 100 kbp away from the most significant SNP marker for ICF (BTA20:66806800, rs384363430). Previously a significant SNP was detected on BTA20 for ICF in Finnish Ayrshire [4]. However with the position of this marker around 4.9 Mb this is not overlapping with the region for FTI identified in this study.

### BTA24

The most significant SNP for FTI (BTA24:31820659, rs137134841) coincided with the most significant SNP for AISC and IFLC. In the same region ranging from 31.1 to 32.1 Mb AISH, IFLH and NRRH shared their most significant SNP (BTA24:31817915, rs381174897). The two SNPs are close to each other in the genome and are located in an intron in the gene *ZNF521. ZNF521* is among others related to protein domain specific binding (GO:0019904). This gene has not been reported to be associated with fertility before, but has been reported as a candidate gene for the trait, rear side view, in Chinese Holstein cattle [24]. Previously significant SNP associations on BTA24 have been identified for ICF on BTA24 in Finnish Ayrshire [4]. These SNPs however are not located in the QTL region detected in this study.

### Intergenic regions

All the significant SNPs on BTA5 and BTA20 were located in intergenic regions. With the improvement of the Bovine annotation of the genes and localization of the set sites of gene transcription, initiation, termination as well as differential splicing one would expect the identification of the causal mutation to improve. To date information on genomic structure of organisms which are better annotated like mouse and human are used as an information source. However it has been shown that areas which contain mainly regulatory elements and non-protein coding regions the annotation are more challenging [25, 26].

Though we have used the “full” sequence level variants in our analysis half of the total genetic variants identified in the WGS were filtered out for various quality control reasons. All the variants which were not bi-allelic were dropped due to limitations in the imputation software. Therefore the actual causal polymorphism may be missing from the data analyzed here.

### Cow versus heifer traits

The genetic correlation between cow and heifer fertility is generally low [27, 28]. This indicates that little overlap in genes for cows and heifers influencing female fertility are expected. The results of this study show that if female fertility traits had their most significant SNP marker in common with the fertility index these traits were either cow traits or heifer traits. This is in agreement with previous QTL mapping studies [6, 29].

## Conclusion

The results of this study showed that 1) the traits AISH and IFLH had in many cases the same significant SNP marker in the QTL detected for FTI, 2) the majority of the candidate genes assigned to the most significant SNP regulating female fertility were involved in protein binding, and 3) a SNP marker on BTA20 located in the QTL region for FTI has previously been validated for female fertility in three different breeds.

### Availability of data

Genome assembly data were taken from publicly available sources. The assembly is available for download (ftp://ftp.ncbi.nlm.nih.gov/genomes/Bos_taurus/). All whole genome sequencing data for RDC animals are now part of the 1000 Bull Genomes Project. All variations detected by the 1000 Bull Genomes Project have been or will be submitted by the 1000 Bull Genomes Project to dbSNP (http://www.ncbi.nlm.nih.gov/projects/SNP/). Whole genome sequence data for the 253 individuals included in Run2 of the 1000 Bull Genomes Project are available at NCBI using SRA no. SRP039339 (http://www.ncbi.nlm.nih.gov/bioproject/PRJNA238491). All annotation information was obtained from a publicly available source (http://www.ensembl.org).

## Additional files

Additional file 1: Figure S1. Plot of the distribution of the accuracy for the Estimated Breeding Values for Fertility Index in Nordic Red Cattle. (PNG 3 kb) Additional file 2: Table S1. SNPs significantly associated with Fertility index in the Nordic Red Cattle. A genome wide association scan was performed on the Nordic Red Cattle for the fertility index. SNPs were considered significant at Bonferroni corrected *P*-value < 0.05 (−log_10_(*P*) > 8.25). (TXT 1811 kb)
