# Supplementary figures and images for: Genome-wide association study for female fertility in Nordic Red cattle
Source: BMC Genet. 2015 Sep 15;16:110. doi: 10.1186/s12863-015-0269-x (PMC4570259; doi:10.1186/s12863-015-0269-x)

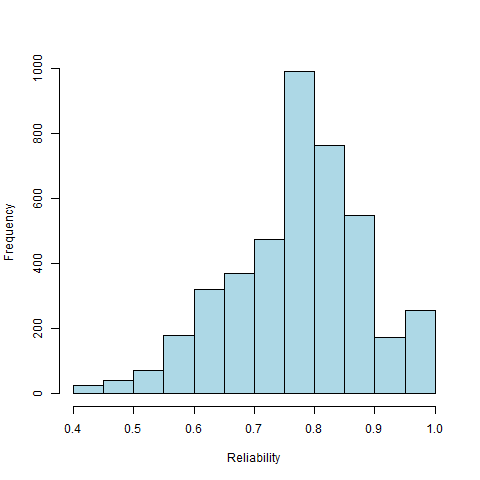

Supplement: Additional file 1: Figure S1. — Plot of the distribution of the accuracy for the Estimated Breeding Values for Fertility Index in Nordic Red Cattle. (PNG 3 kb) [file 12863_2015_269_MOESM1_ESM.png]
